# The potential of miRNA-based approaches in glioblastoma: An update in current advances and future perspectives

**DOI:** 10.1016/j.crphar.2024.100193

**Published:** 2024-06-29

**Authors:** Edgar G. Ordóñez-Rubiano, Nicolás Rincón-Arias, Sebastian Espinosa, William J. Shelton, Andres F. Salazar, Alba Cómbita, Matías Baldoncini, Sabino Luzzi, César Payán-Gómez, Diego F. Gómez- Amarillo, Fernando Hakim, Javier G. Patiño-Gómez, Rafael Parra- Medina

**Affiliations:** a School of Medicine, Universidad Nacional de Colombia, Bogotá, Colombia; b Department of Neurosurgery, Fundación Universitaria de Ciencias de La Salud, Hospital de San José – Sociedad de Cirugía de Bogotá, Bogotá D.C., Colombia; c Department of Neurosurgery, Fundación Santa Fe de Bogotá, Bogotá, Colombia; d School of Medicine, Universidad de Los Andes, Bogotá, Colombia; e Department of Microbiology, Universidad Nacional de Colombia, Bogotá, Colombia; f School of Medicine, Laboratory of Microsurgical Neuroanatomy, Second Chair of Gross Anatomy, University of Buenos Aires, Buenos Aires, Argentina; g Department of Neurological Surgery, Hospital San Fernando, Buenos Aires, Argentina; h Neurosurgery Unit, Fondazione IRCCS Policlinico San Matteo, Pavia, Italy; i Dirección Académica, Universidad Nacional de Colombia, Sede de La Paz, La Paz, Colombia; j Department of Pathology, Instituto Nacional de Cancerología, Bogotá, Colombia; k Research Institute, Fundación Universitaria de Ciencias de La Salud (FUCS), Hospital de San José – Sociedad de Cirugía de Bogotá, Bogotá, Colombia

**Keywords:** microRNA, miRNA, RNA, mRNA, Glioblastoma, Glioma

## Abstract

Glioblastoma (GBM) is the most common malignant central nervous system tumor. The emerging field of epigenetics stands out as particularly promising. Notably, the discovery of micro RNAs (miRNAs) has paved the way for advancements in diagnosing, treating, and prognosticating patients with brain tumors. We aim to provide an overview of the emergence of miRNAs in GBM and their potential role in the multifaceted management of this disease. We discuss the current state of the art regarding miRNAs and GBM. We performed a narrative review using the MEDLINE/PUBMED database to retrieve peer-reviewed articles related to the use of miRNA approaches for the treatment of GBMs. MiRNAs are intrinsic non-coding RNA molecules that regulate gene expression mainly through post-transcriptional mechanisms. The deregulation of some of these molecules is related to the pathogenesis of GBM. The inclusion of molecular characterization for the diagnosis of brain tumors and the advent of less-invasive diagnostic methods such as liquid biopsies, highlights the potential of these molecules as biomarkers for guiding the management of brain tumors such as GBM. Importantly, there is a need for more studies to better examine the application of these novel molecules. The constantly changing characterization and approach to the diagnosis and management of brain tumors broaden the possibilities for the molecular inclusion of novel epigenetic molecules, such as miRNAs, for a better understanding of this disease.

## Highlights

• miRNAs with their 18–22 base pairs, play a crucial role in regulating gene expression through pre- and post-transcriptional mechanisms in glioblastomas, influencing mRNA degradation.
• miRNAs are present in the cerebrospinal fluid and are considered potential candidates for biomarkers associated with molecular diagnosis and prognosis in glioblastomas.
• MiR-21 and miR-let-7 are linked to oncogenic and tumor-suppressive functions: influencing critical pathways like PI3K/AKT and RAS signaling in glioblastomas.
• miRNAs have shown potential as a tool in the molecular diagnosis, treatment, and prognosis of glioblastomas. Continuous research efforts are crucial to advance new treatments with accurate molecular tools.

## Graphical abstract

**Figure:**
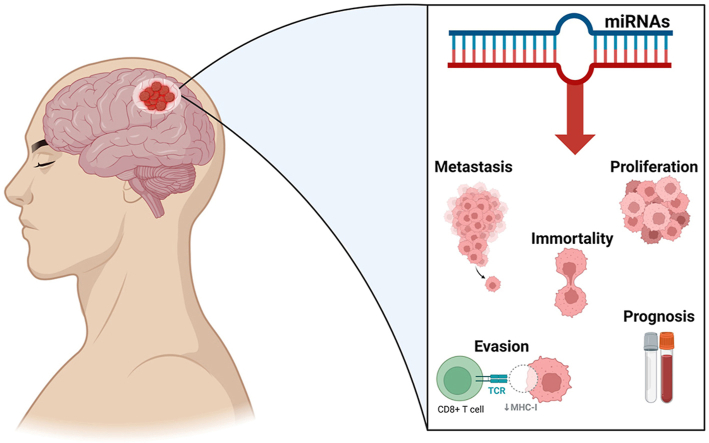

## 1 Introduction

Gliomas stand out as the most common malignant primary central nervous system (CNS) tumor (Louis et al., 2021). Glioblastoma (GBM) is the most prevalent glioma and is classified as a grade 4 tumor according to the 5th edition of the World Health Organization (WHO) classification (WHOCNS5) (Louis et al., 2021). It accounts for 14.5% of all CNS tumors and 48.6% of all malignant CNS tumors (Grochans et al., 2022). Despite the availability of multimodal therapeutic approaches, GBM still exhibits a poor prognosis with a five-year survival rate of 5.5% (Ostrom et al., 2017). Data has shown how, over the past 30 years, the median survival of GBM has not changed significantly, with a low median survival rate of 2 years or less (Liu et al., 2013). Furthermore, the life expectancy of patients with GBM is approximately 1 year, and for patients exhibiting recurrence is around 4 months (Zhao et al., 2017a). Although there has been significant progress in the comprehension of GBM biology, there is still a conceptual gap concerning the molecular mechanisms responsible for pathogenesis and therapeutic options for treating this disease (Gonzalez-Gomez et al., 2011; Low et al., 2014). However, recent advancements in molecular pathology have unveiled compelling links between glioma development and various epigenetic phenomena involving histone modifications, deoxyribonucleic acid (DNA) methylation, chromatin remodeling, and dysregulation of ribonucleic acid (RNA) profiles (Phillips et al., 2020; Uddin et al., 2022). These advances, which have led to different approaches with numerous and novel therapeutic strategies, such as gene editing, epigenetic drugs, or micro RNA (miRNA) modifications, have molded a path for reducing the pathological impact of this disease (Uddin et al., 2022).

Recently, non-coding RNAs (ncRNAs), such as miRNAs, have emerged as new effectors in the epigenetic field, capable of influencing gene transcription and translation without altering the DNA sequence, as traditionally seen in other epigenetic processes. (Banelli et al., 2017) miRNAs play crucial roles in regulating cell cycle checkpoints and tyrosine signaling pathways (Ames et al., 2017). The significance extends to the regulation of cancer, (Beylerli et al., 2022) neural development, (Ma et al., 2023; Nowakowski et al., 2018; Ivey and Srivastava, 2015) and stem cell functions (Gangaraju and Lin, 2009). For example, miR-21 and miR-26 are overexpressed in GBM, which act on mRNA of many genes related to P53, a well-known tumor suppressor and transcription factor, directly related to cell cycle arrest (Chan et al., 2005). Consequently, the decreased expression of these miRNAs can inhibit cell cycle arrest and cell death (Sati and Parhar, 2021). On the other hand, miRNAs can also regulate the retinoblastoma (RB) pathway. MiR-124 and miR-137 are downregulated in GBM. Restoring their normal expression levels increases cell cycle arrest at the G0/G1 phase (Silber et al., 2008; Godlewski et al., 2008). These expressions are related principally to the regulation of the cyclin-dependent kinases (CDK) signaling pathways. On the other hand, recent studies have identified a specific subset of cancer stem cells (CSCs) within solid tumors like GBM (Piper et al., 2021; Lathia et al., 2015). These CSCs can initiate tumor growth, drive malignant progression, and confer resistance to radiation and chemotherapy. Notably, GBM-derived CSCs share essential characteristics with neural stem cells (NSCs), such as self-renewal and multipotency, which may be influenced by miRNAs (Makowska et al., 2023; Khan et al., 2019). Importantly, the upregulation of miR-21 is the most pronounced within high-grade gliomas (HGGs) (Aloizou et al., 2020; Nieland et al., 2022; Belter et al., 2016). Conversely, a reduction in the expression levels of both miR-219 and miR-7 has been associated with an elevation in the expression of the epidermal growth factor receptor (EGFR), a receptor tyrosine kinase commonly observed to be overexpressed and activated in GBM (Ames et al., 2017). Here we aim to provide an overview of the current understanding of miRNAs in GBM development, with a focus on the current advances in diagnosis and treatments as well as future perspectives.

## 2 Materials and methods

A comprehensive narrative review of the latest available literature was done regarding the current use of miRNA in GBM in both English and Spanish languages. A focus was made on pathophysiology, diagnosis, prognosis, and treatment. The search was done by screening titles and abstracts of pertinent articles using the MEDLINE/PUBMED database. References were inspected for gathering additional studies. Schematic illustrations were also included.

We also performed a scoping review regarding the role of miRNAs in liquid biopsies for GBM detection and how such diagnostic tools could significantly enhance therapeutic strategies for managing GBM patients clinically. We reviewed all original studies indexed in PUBMED and EMBASE databases published in English and Spanish. The search included data from 2008 to 2024. The screening guidelines encompassed studies with fundamental demographic data, and follow-up information, and were accessible via these databases. The databases were last consulted on May 21, 2024. Our review included 1924 studies. The abstracts were reviewed and filtered by WJS, AFS, EGO, and NRA. Only original studies were included. A total of 44 were finally incorporated into the review. Data from the articles in the review was extracted using an artificial intelligence (AI) platform (*TextCortex* [https://textcortex.com/pdf-ai-alternative]). Once the selected articles were obtained in PDF format, the AI submitted and processed them to identify and retrieve specific miRNAs mentioned concerning glioblastoma. The AI was instructed to extract details on the miRNAs' associations with glioblastoma, including the biological fluids in which they were found and their reported utilities.

## 3 Overview

### 3.1 Biogenesis of miRNA

**Fig. 1:**
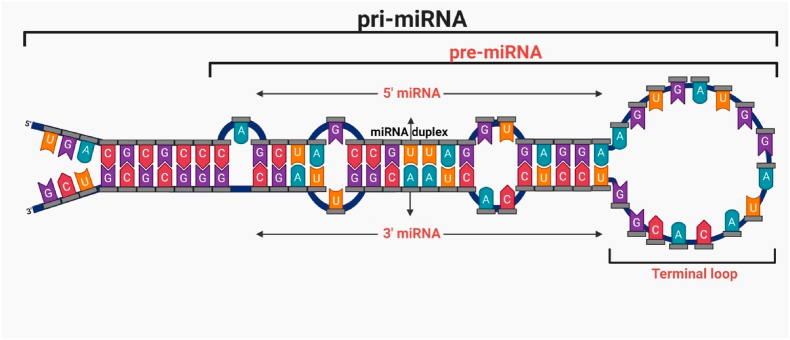
**Illustration of the structure of a miRNA.** The illustration is depicting the biogenesis process of miRNA molecules from a pri-miRNA to a pre-miRNA and finally to a mature miRNA, represented as a duplex.

**Fig. 2:**
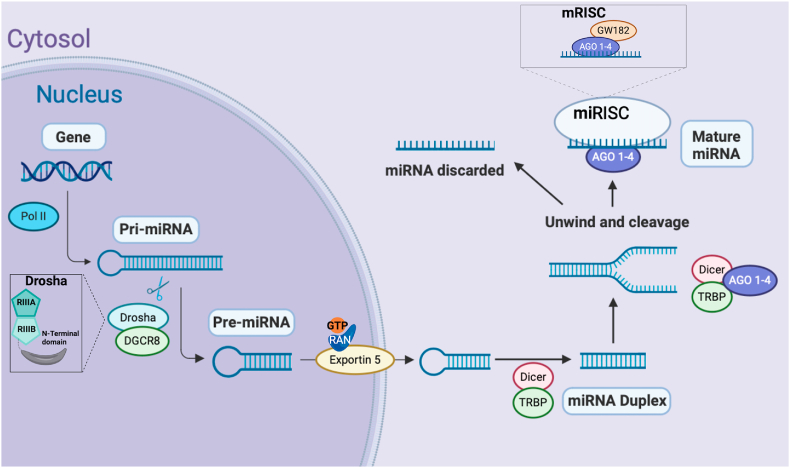
**Biogenesis of miRNAs.** In the nucleus, the genes that code for miRNAs are transcribed in the form of long precursors, giving rise to the so-called primary miRNAs (pri-miRNAs), whose length varies between hundreds of pairs of nucleotides. This precursor is cut by Drosha/DGCR8 ribonucleases into one or several hairpin-shaped RNA molecules, transforming it into pre-miRNAs of 60–70 nucleotides. Drosha is composed of two RNAase III domains (RIIIA and RIIIB) and an N-terminal domain. The pre-miRNAs leave the nucleus towards the cytoplasm helped by Exportin 5 (a RANGTP-dependent binding protein), where the miRNA maturation process will take place. In the cytoplasm, the pre-miRNA is transported by the RLC complex (microRNA-induced silencing complex [miRISC] loading complex) where the RNAase Dicer/TRBP acts. This complex produces the cleavage of the pre-miRNA, generating a duplex miRNA with a mature miRNA chain and its complementary one. The mature strand together with AGO 1–4 and WG182 will form the miRISC and the complementary strand will be eliminated. MiRISC binds to an mRNA molecule (usually in the 3′ untranslated region) that has a sequence complementary to its miRNA component and cleaves the mRNA, leading to degradation of the mRNA or modification of its translation. Image created with www.biorender.com.

MiRNAs constitute a class of intrinsic ncRNAs with approximately 18–22 base pairs in length (Fig. 1), playing a crucial role in regulating gene expression through pre- and post-transcriptional mechanisms, particularly messenger RNA (mRNA) degradation (Chen et al., 2021; Bartel, 2004; Xiao et al., 2017). The biogenesis of miRNAs is illustrated in Fig. 2. These molecules modulate gene expression by interacting with the 3′-untranslated region (3′-UTR) of target mRNAs (O'Brien et al., 2018). Functioning through non-mutational mechanisms, miRNAs serve as significant epigenetic effectors (Banelli et al., 2017). Additionally, it is important to note the importance of epigenetics in the biogenesis of miRNAs. This field, which refers to the study of the variations in gene expression due to genetic alterations, (Farsetti et al., 2023) is known for its different reversible and heritable processes involving DNA methylation, histone modifications, and various RNA-mediated changes (Zhang et al., 2020). Epigenetic mechanisms such as DNA methylation and histone modifications influence the transcriptional control of miRNA expression. For example, for miR-127, the methylation of the CpG sites and deacetylation of the histones contribute to its silencing in tumor cell lines (Chuang and Jones, 2007).

### 3.2 miRNAs as biomarkers in GBM

The significance of biomarkers primarily lies in their ability to identify specific tumor treatments and monitoring of diseases, which is primarily done with a tumor biopsy. However, in cases such as GBM, this is not always feasible given the high risks of neurological decline of performing a new intervention if the tumor is located deeply or near to or within an eloquent area (Freidlin and Korn, 2014). Traditional biomarkers such as methyl-guanine-methyl-transferase (MGMT) which are associated with better prognosis and increased sensitivity to alkylating agents such as temozolomide (TMZ), still pose uncertainties in comparison to other molecular markers. The persistence of low survival rates in GBM over time underscores the need for developing new prognostic biomarkers that could aid in clinical decision-making (Huang et al., 2018). Consequently, considering that miRNAs are present in most body fluids, they have been considered potential candidates to serve as biomarkers for various pathologies (Weber et al., 2010). For instance, in gliomas, miRNAs have been described as possible biomarkers that could be associated with prognosis, prevention, or progression of the disease, as well as with the response to adjuvant treatments (Mucaj et al., 2015; Que et al., 2015).

### 3.3 miRNAs expression profiles in GBM

**Fig. 3:**
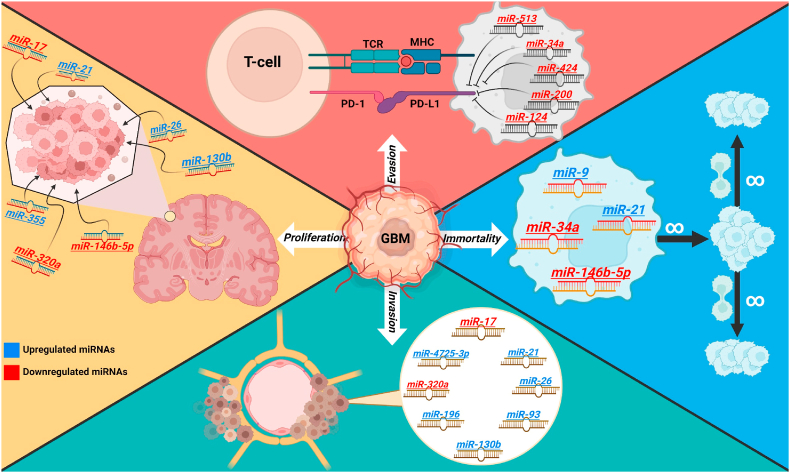
**MiRNA mechanisms in the pathogenesis of GBM.** miRNAs play a fundamental role in GBM through the upregulation or downregulation of essential cellular processes, resulting in cell immortality, uncontrolled cell proliferation, immune evasion, and brain invasion. The figure depicts examples of miRNAs known to be involved in these processes, with arrows indicating upregulation and downregulation. Image created with www.biorender.com.

The use of bioinformatic methods (e.g., clustering) and miRNA expression profiling has been shown to produce a better classification of tumors in terms of histology and prognosis than the sole use of mRNA expression (Huang et al., 2018). The role of miRNAs in cancer biology, including GBM, has been widely explored (de Menezes et al., 2021). One of the most studied miRNAs is miR-21, which is increased in many cases of GBM and appears to act as an oncogene (Brower et al., 2014). Similarly, miR-let-7 is often overexpressed, and this overexpression has been related to a decrease in cellular invasion and migration rates (Kong et al., 2012). Different deregulated miRNAs that are related to GBM are summarized in Table 1 (de Menezes et al., 2021; Bendahou et al., 2020). Additionally, some of them have been identified as possible treatment targets: miR-9, miR-21, miR-7, miR-34a, miR-4492, miR-320a, miR-146 b-5p, miR-320 A, and miR-146 b (de Menezes et al., 2021). On the other hand, different miRNAs have been related to the epithelial-mesenchymal transition (EMT) (Setlai et al., 2022a). The expression of several ligands binding to tyrosine kinase receptors is influenced by specific miRNAs. For instance, the phosphatase and tensin homolog (PTEN) gene, encoding a tumor suppressor protein that negatively regulates the PI3K/AKT signaling pathway and thus controls cellular proliferation, is negatively regulated by miR17–5p, miR-23a-3p, and miR-26a-5p (Ghafouri-Fard et al., 2021; Mukherjee et al., 2009). Similarly, the RAS signaling pathway, which is associated with cancer development by the upregulation of oncogenic transcription, increasing cell motility, survival, growth, metabolism, and migration, is upregulated by miR-143–3p, miR-123–3p, and let5a-5p (Setlai et al., 2022b; Gimple and Wang, 2019). Even critical tumor suppressors, like the p53 gene, are regulated by miRNAs such as miR10p-5p (Setlai et al., 2022b). Additionally, exosomal miRNAs contribute to the understanding of GBM, as some are released during disease progression (miR-21, miR-301, miR-301a) (Aili et al., 2021). These exosomes can release miRNAs to surrounding normal cells through endocytosis or lipid membrane fusion, disrupting the homeostasis of normal cells, and promoting the proliferation and invasion of malignant cells (Aili et al., 2021). Compared to exosomes derived from normal brain tissue, exosomes derived from tumor cells exhibit significantly increased expressions of miR-222, miR-9, and miR-26a, activating numerous signal transduction pathways to stimulate tumor growth (Riches et al., 2014). Some of these exosomal miRNAs include: miR-301a, (Yue et al., 2019) miR-151a, (Zeng et al., 2018) miR-21, (Shi et al., 2015) miR-1246, (Guo et al., 2016) miR-29a, (Guo et al., 2019) miR-92a, (Guo et al., 2019) miR-9, (Riches et al., 2014) miR-26a, (Kim et al., 2010) and miR-375, (Li et al., 2019) among others (Aili et al., 2021). Some of the miRNAs involved in cellular processes in GBM are illustrated in Fig. 3.

**Table 1:** Expression of miRNAs involved in the molecular pathways of glioblastomas.

miRNA	Expression	Target	Pathway involved	Effect in GBM
miR-7 (de Menezes et al., 2021)^,^ (Matos et al., 2018)	Decreased		Normally inhibits epidermal growth factor	Related to greater invasion and worse post-treatment prognosis.
miR-9 (Ben-Hamo et al., 2016)	Elevated	P38	Protein kinase pathway, SRF, CREB1, YWHAZ, MAPKAPK2	Stress, differentiation, and cell progression
miR-17 (Wang et al., 2014)	Decreased	EGFR, IRS1, IRS2	IGF-1R/Akt	Invasion, proliferation
miR-21 (Matos et al., 2018)	Elevated	LRRFIP1, STAT3, BAX/Bcl‐2/caspase 3	ARF-MDM2-P53	Invasion, proliferation, migration and cell cycle
miR-25 (Birks et al., 2011)	Decreased	MDM2-TSC1	ARF-MDM2-P53	Regulation of intrinsic and extrinsic mediated apoptosis. Tumor suppressor pathways.
miR-26 (Huse et al., 2009)	Elevated	P53	ARF-MDM2-P53	Invasion, proliferation, migration and cell cycle
miR-30 b-3p (Ahn et al., 2018)	Decreased	PIK3CD, CX3CL1	Proteoglycans, cytokine-cytokine receptor pathway interaction.	Progression and pathogenesis
miR-32 (Bao and Li, 2019)	Decreased	MDM2-TSC1	ARF-MDM2-P53	Regulation of intrinsic and extrinsic mediated apoptosis. Tumor suppressor pathways.
miR-34a (Toraih et al., 2018; Sun et al., 2008; Yin et al., 2013)	Decreased	GAS5, cyclinE2, CDK4/6, p53	Proteoglycans, adherent junctions, cell cycle, P53 signaling. CDKs-RB-E2F, ARF-MDM2-P53.	Cell death, cell cycle, cell cycle arrest, cell motility, invasion, metastasis.
miR-93 (Fang et al., 2011)	Elevated			Tumor invasion, poor prognosis
miR-124 (Godlewski et al., 2008)	Decreased	CDK6, cyclinD1	CDKs-RB-E2F	Cell cycle arrest
miR-126 (Wu et al., 2021)	Decreased	P13K-Akt	ARF-MDM2-P53	Regulation of intrinsic and extrinsic mediated apoptosis. Tumor suppressor pathways.
miR-128 (Zhang et al., 2009)	Decreased	E2F3a	CDKs-RB-E2F	Cell cycle arrest
miR-130 b (Gu et al., 2016)	Elevated	PPAR-γ	PPAR-γ/β‐catenin	Cell proliferation and invasion
miR-137 (Godlewski et al., 2008)	Decreased	CDK6	CDKs-RB-E2F	Cell cycle arrest
miR-146 b-5p (Liu et al., 2015)	Decreased	TRAF6	TRAF6-TAK1	Cell proliferation and resistance to apoptosis
miR-181 b (Slaby et al., 2010)	Decreased	MGMT	DNA repair	O (6)-Methylguanine-DNA-methyltransferase (MGMT) is a unique protein which repairs. Determinates therapeutic success of alkylating agent chemotherapy, specifically temozolomide treatment.
miR-181c (Ayala-Ortega et al., 2016)	Decreased	NOTCH2, MGMT	NOTCH signaling, DNA repair	Tumor progression. Determinates therapeutic success of alkylating agent chemotherapy, specifically temozolomide treatment.
miR-196 (Chen et al., 2011)	Elevated			Cellular proliferation, poor prognosis
miR-203 (Chen et al., 2017a)	Decreased	TS mRNA	miR-203-TS	TMZ resistance, senescence and cell cycle arrest
miR-320 (Manterola et al., 2014)	Elevated		Acts in conjunction with miR-574-3 as possible biomarkers	
miR-320a (Li et al., 2017)	Decreased	SND1, β‐catenin	TGFβ1 pathway	Cell proliferation, invasion and migration
miR-355 (Zhi et al., 2017)	Elevated			Cell proliferation
miR-375 (Deng et al., 2020)	Decreased	SLC31A1	SLC31A1-MMP9	Copper cellular transport, compartmentalization and incorporation. Matrix metalloproteinase-9 regulator and effecter of cellular processes.
miR-874–5p (Ahn et al., 2018)	Decreased	PIK3CD, CX3CL1	Proteoglycans, cytokine-cytokine receptor pathway interaction	Progression and pathogenesis
miR-4492–3p (Ahn et al., 2018)	Elevated	COL2A1, CNR1	Rap 1 pathway	Progression and pathogenesis
miR-4725–3p (Ho et al., 2018)	Elevated	STM1	SOCE pathway	Cell invasion and tumor suppressor

Many miRNAs influence tumor pathways such as in GBM, ultimately modifying the regulation of mRNA in terms of their genetic expression (Chen et al., 2021). This is the case for the widely studied miR-21 which has been identified as an apoptotic regulator as demonstrated in studies where knockdown of the molecule resulted in cell apoptosis via caspase activation (Chan et al., 2005). By targeting several proteins such as Tap 63, Heterogeneous Nuclear Ribonucleoprotein K (HNRPK), and Programmed Cell Death Protein 4 (PDCD4), miR-21 achieved inhibition of apoptotic pathways, hence further contributing to tumor cell proliferation (Chen et al., 2021). Cell proliferation on the other side, has been linked to direct action of miR-21 on PTEN, SMARCA4, and ANP32A genes among others (Kwak et al., 2011; Schramedei et al., 2011). On the other hand, multiple miRNAs have been identified to target oncogenes and play tumor suppressive roles as is the case for miR-7 (downregulated in GBM) by targeting PI3K and Raf-1 via the EGFR pathway, (Liu et al., 2014) and miR-128 was found to decrease glioma cell proliferation by targeting E2F3a (Zhang et al., 2009). Several studies have also highlighted the fundamental role of different subtypes of miRNAs in CNS tumor development (Zhang et al., 2012). A study evaluated the invasion potential of miR-221/222 using methods such as diffusion tensor imaging, transwell assay, wound healing, and mouse tumor xenograft assays. In this study, the knockdown of miR-221/222 correlated with decreased cell invasion by interfering with tissue inhibitor of metalloproteinases (TIMP3) levels (Zhang et al., 2012). Additionally, miR-221/222 knockdown was shown to inhibit tumor growth by increasing TMP3 expression.

Importantly, miRNAs regulate crucial genes that play a fundamental role in the various pathways related to tumorigenesis (Uddin et al., 2022). For example, in CNS tumors, miRNAs have been shown to control glioma stem cell differentiation and tumor development (Sana et al., 2018; Mahinfar et al., 2022). Several studies have highlighted the fundamental role of different subtypes of miRNAs in CNS tumor development (Zhang et al., 2012). A study done by Zhang et al. evaluated the invasion potential of miR-221/222 using methods such as diffusion tensor imaging, transwell assay, wound healing assay, and a mouse tumor xenograft assay. In this study, the knockdown of miR-221/222 correlated with decreased cell invasion by interfering with tissue inhibitor of metalloproteinases (TIMP3) levels (Zhang et al., 2012). Additionally, miR-221/222 knockdown was shown to inhibit tumor growth by increasing TMP3 expression.

### 3.4 miRNAs-based GBM classifications

While mRNA-based classifications for GBM exist, they have not gained widespread acceptance, primarily because miRNAs have demonstrated greater accuracy in classifying and diagnosing tumor samples compared to mRNAs and because they have provided more accurate and significant demographic data and clinical information regarding prognosis. MiRNA cluster identification has allowed glioblastoma typification into five subclasses related to its tumor cell precursor. Five clusters have been identified allowing for a differentiation-related classification system of glioblastoma into five subclasses: oligoneural, neural, astrocyte, neuro mesenchymal, and radial glial precursors subtypes. Each of these suggests a relationship between each subclass and a distinct stage of neural differentiation (Kim et al., 2011). When comparing subtypes based solely on RNA expression, oligoneural precursors correspond to the proneural GBM subtype due to mutations in isocitrate dehydrogenase 1 (IDH1), mesenchymal neural precursors correspond to the mesenchymal GBM subtype due to mutations in NF1, and radial glial may correspond to the classic GBM subtype due to high levels of EGFR. However, GBM classification becomes more intricate when considering the cell subtypes of each tumor, their mixed cellular states (as GBM stem cell subpopulations maintain transcriptomic heterogeneity), and even the neural differentiation stage at which the tumor cell was developed (Huang et al., 2018).

As aforementioned, these precursor-related subclasses are associated with demographic characteristics and prognosis showing cluster associations with race, age, treatment response, and patient survival rates. As shown by Kim et al. when compared with astrocytic tumors, patients with neuro mesenchymal glioblastomas exhibited a trend towards longer survival. Additionally, patients with oligoneural glioblastomas had a notably longer survival time compared to those with radial glial, neural, or astrocytic tumors. On average oligoneural glioblastomas were noted to be diagnosed in younger patients and racial differences across the miRNA-based glioblastoma subclasses, with a higher percentage of non-Caucasian patients found in the neural and astrocytic subclasses compared to the radial glial subclass (Kim et al., 2011). These miRNA clusters could potentially serve as biomarkers for diagnosis, aiding in further classification of these tumors and providing prognostic information.

When comparing subtypes based solely on RNA expression, oligoneural precursors correspond to the proneural GBM subtype due to mutations in isocitrate dehydrogenase 1 (IDH1), mesenchymal neural precursors correspond to the mesenchymal GBM subtype due to mutations in NF1, and radial glia may correspond to the classic GBM subtype due to high levels of EGFR. However, GBM classification becomes more intricate when considering the cell subtypes of each tumor and their mixed cellular states, as GBM stem cell subpopulations maintain transcriptomic heterogeneity (Huang et al., 2018). Furthermore, genetically distinct subclasses are observed based on differences in race, age, treatment response, and patient survival rates. A study done by Kim et al. on 121 selected miRNAs, revealed a highly varied expression closely related to patient survival or previously associated with neuronal development (Kim et al., 2011). Additionally, the presence and deregulation of miRNAs in blood or cerebrospinal fluid (CSF) could potentially serve as biomarkers, such as miR-21 (Zhou et al., 2018).

### 3.5 microRNAs: dynamic interaction of pro-oncogenic vs anti-oncogenic functions in GBM

MiRNAs play a significant role in the regulation of gene expression. They have been proven to be important regulators of gene expression and are involved in modulating many cellular processes including apoptosis, proliferation, invasion, angiogenesis, and chemoresistance in GBM (Chen et al., 2021). Hence alterations in expression and function of different miRNAs contribute to the complex molecular landscape of the disease. The level of individual miRNAs can present different dynamic changes at various stages of the development of a tumor. It is important to consider the miRNA profile in GBM because it indicates the stage of the disease and can be in relationship with the prognosis and selection of an appropriate therapy. (Makowska et al., 2023) miRNAs can act both as anti- and pro-oncogenic factors by down or upregulating tumor-involved genes. Additionally, the functional analysis of different and GBM-specific miRNAs indicates which act as oncogenes or tumor suppressors and are responsible for developing resistance to chemotherapy and radiotherapy, stimulating neo-angiogenesis and cell proliferation, and regulating the cell cycle and apoptosis (Makowska et al., 2023). According to their roles in tumorigenesis, they can either be classified into tumor suppressors or tumor promoters or can act as both. Tumor suppressor miRNAs target oncogenes, meaning that their decreased expression is involved in the promotion of tumor progression given that tumorigenesis is not inhibited. Generally, those that disrupt the activity of the histone methyltransferase EZH2 can be regarded as tumor suppressors (Paskeh et al., 2022). Notably, miR-let-7 is one such miRNA that not only inhibits EZH2 but targets oncogenes like MYC and K-RAS, enhancing its tumor-suppressive properties (Chirshev et al., 2019). Well-studied tumor suppressor miRNAs include miR-7, miR-34, and miR-128. MiR-7 is downregulated in GBM leading to proliferation, migration, invasion, and metastasis of GBM by allowing overexpression of different oncogenes through the EGFR pathway. Both miR-34 and miR-128 are downregulated, being the latter involved in inhibition of self-renewal of glioma stem cells, and attenuating the effects of cell proliferation, tumor growth, and angiogenesis. MiR-34 on the other hand, induces apoptosis and inhibits cell migration, proliferation, and angiogenesis (Chen et al., 2021). Aside from these tumor suppressors, onco-miRNAs will be involved in the development of GBM by targeting the expression of tumor suppressor genes promoting oncogenesis.

Onco-miRNAs will be upregulated hence promoting GBM progression. The most important onco-miRNAs are miR-10 b, miR-21, and miR-93. MiR-10 b has been implied in the development of HGGs by enhancing the invasive capabilities of the tumor. It has been well documented that a decrease in expression of miR-10 b results in the reduction of cell growth, invasion, and angiogenesis as well as an increase in apoptosis through many mechanisms that involve targeting of RhoC, uPAR, and HOXD10 genes. MiR-21, being the most widely investigated miRNA, has been shown to influence cell invasion, metastasis, and resistance to chemotherapeutics (Chen et al., 2021). It has been identified as an apoptotic regulator with high expression in GBM cells through intricate mechanisms that involve HNRPK, TAp63, FASL, P53, TGF-B, and PDCD4 genes (Chan et al., 2005). Cell proliferation and chemoresistance are also made possible by miR-21 through targeting of specific genes such as MMPs, Ras/Raf, ERK, RECK, and TIMP3 (Chen et al., 2021). Finally, there is evidence that miR-93 is also a critical target in GBM founding to be upregulated in the development of the disease and involved in proliferation, migration, and invasion by affecting cell cycle arrest and promoting angiogenesis through targeting integrin-β8 (Fang et al., 2011).

On the other hand, GSK-3β acts like a potent tumor suppressor of the Wnt/β-catenin axis, due to inhibition of Wnt signaling through targeting β-catenin. Several studies indicate that regulatory miRNAs can also inhibit the axis WNT due to the promoting GSK-3β activity in diverse groups of cancer cells. For example, the tumor suppressor miR-34a has been reported to be downregulated in patients with GBM resulting in poor prognosis and a shorter survival rate (Rahmani et al., 2023).

Some studies have documented in vitro that let-7 acts like a tumor suppressor gene and inhibits the malignant behavior of glioma cells and stem-like cells. However, it is necessary to elucidate many mechanisms of interactions. Additionally, regulation of RAS protein level and RAS/MAPK cascade are regulated by various miRNAs without a clear mechanism (Messina, 2024).

Each miRNA can modulate the expression of several miRNAs, creating an extraordinarily complex regulatory network where different miRNAs can be modulated by several other miRNAs. These biomarkers work as an intricate system of modulation and feedback that can serve both as diagnostics and potential therapeutics (Chen et al., 2021). It is therefore indispensable to understand this miRNA biology in order to continue identifying the emergent and continuous number of miRNAs with their corresponding targets for developing novel molecular therapies and diagnostic methods for better treatment of GBM.

### 3.6 How can microRNAs be important in future diagnosis and treatments?

Current diagnosis and treatment of GBM represent a challenge that requires an integrated approach combining histologic, molecular, and imaging information. Classification and grading of these tumors were once entirely based on morphological parameters such as pleomorphism, angiogenesis, presence of necrosis, and mitotic activity. Parameters that carried important limitations given tumoral heterogeneity at multiple levels, including genomic, morphological, cellular, clinical, and functional ones (Balana et al., 2022). Also, technical limitations such as sampling errors, both of which imply a high variability in diagnosis and therefore, treatment.

With the arrival of molecular characterization of gliomas, grading became more specific, impacting patient prognosis, improving treatment planning, and reducing diagnostic variability making molecular analysis crucial in the management of these entities. More recently, the WHOCNS5 has incorporated several molecular biomarkers (IDH1/2 mutation, 1p19q co-deletion, MGMT methylation, etc.) that have aided in the definition of both grade and histological subtypes of diffuse gliomas (Balana et al., 2022).

For example, the WHOCNS5 has classified diffuse gliomas into IDH mutant and IDH-wildtype tumors, making identifying and guiding further molecular classification easier. IDH mutant tumors include oligodendrogliomas (expressing a 1p/19q codeletion), astrocytomas, IDH mutants, grade 2 and 3 (expressing P53 and ATRX mutations), and astrocytomas, IDH mutants, grade 4 (expressing the CDKN2A/B mutation). On the other side, IDH wildtype gliomas include astrocytomas, IDH wildtype, grade 2 and 3, and GBM (expressing TERT or EGFR mutations, or gain of chromosome 7 and loss of chromosome 10). This impacts directly not only on a better characterization and classification of tumors into different entities but also provides information on the impact on survival (Louis et al., 2021; Rubiano et al., 2023).

On the other hand, imaging, which was once considered the cornerstone of glioma diagnosis, has somewhat diminished in importance due to factors such as interobserver variability heterogeneity and tumor presentation heterogeneity. Despite advancements in diagnostic radiology, imaging still falls short in detecting molecular and cellular changes, limiting its ability to accurately identify tumor types (Khristov et al., 2023). However, this technology is hindered by its limited role in the evaluation of therapeutic response, showing limited utility when differentiating complete or partial response to therapy, and stable or progressive disease (Shankar et al., 2017).

GBM's high heterogeneity is a hindrance to diagnosis and hence an adequate treatment that targets molecular therapeutic needs. Considering the limitations of current diagnostic methods for GBM, (Skouras et al., 2023) there is an emphatic need to identify novel methods that in the context of a molecular era, contribute to the idea of finding additional molecular biomarkers that can aid in early diagnosis while preventing invasive diagnostic strategies such as the current tissue biopsy approach. Both to avoid complications, and to properly classify patients early in the disease providing an adequate molecular characterization, prognosis, and oriented therapy (Saenz-Antonanzas et al., 2019). Given this, less invasive methods are becoming increasingly attractive, such as liquid biopsy as a diagnostic option, which, although continues to be studied, has provided a favorable and innovative panorama in the diagnosis of GBM.

Upon directing attention toward neoplastic diseases, biomarkers can be grossly classified into two classes: tumor-derived biomarkers and tumor-associated biomarkers. Both of which have proven to serve to identify both disease presence and progression. The former type is directly related and traced to the tumor, while associated biomarkers appear in response to the disease state of the body (Khristov et al., 2023). Body fluids, particularly blood and its components and CSF, being in close contact with the central and deep structures of the CNS, serve as a diffusion platform for local transport of products derived from neoplasms that ultimately end up representing the biomarkers mentioned above.

### 3.7 The use of miRNAs in liquid biopsies for GBM detection

Liquid biopsy, primarily through blood tests, involves the detection and quantification of tumoral content released into biofluids. Different circulating biomarkers have been proposed for GBM, in particular circulating DNA (ctDNA), and circulating cell-free tumor RNA (ctRNA) that includes mRNAs, lncRNAs, and mainly small non-coding RNAs (sncRNAs). SncRNAs include in turn miRNAs, small interfering RNAs (siRNAs), circular RNAs (circRNAs), small nuclear RNAs (snRNAs), and small nucleolar RNAs (snoRNAs). Among them, miRNAs have arisen as promising biomarkers for cancer diagnosis in the last decade, since they have unique characteristics that make them suitable for isolation. MiRNAs are remarkably stable in plasma and serum, given that they are resistant to RNAase activity, (Garcia and Toms, 2020) and they are the most abundant circulating free molecules in the blood. Also, detectable miRNA levels can be observed in additional cell-free body fluids as well as in tissues. As miRNAs are directly derived from cells serving as important regulatory molecules, altered miRNA expression patterns in biological fluid samples will correlate with tumor presence, providing information on tumoral response to therapy, relapse of the disease, and progression. As has been proposed previously, altered miRNA expression patterns in biological fluid samples correlate with tumor tissue samples, volume, functional performance status, and even prognosis (Saenz-Antonanzas et al., 2019).

MiRNAs can be found either free within serum or CSF or locked within lipid membranes known as exosomes, (Garcia and Toms, 2020) serving as regulatory molecules that affect signal transduction pathways involved in cellular proliferation and suppression by either promoting or suppressing apoptosis (Ahmed et al., 2021). Exosomes are membrane-enclosed extra-cellular vesicles (EVs), that are actively released by both healthy cells and cancer cells carrying nucleic acids (mRNA, DNA, non-coding RNA), lipids, and proteins. These exosomes released by cancer cells can be extracted as non-invasive, circulatory biomarkers containing molecular characteristics of the original tumor and can be screened for detecting these signatures (Makowska et al., 2023).

**Fig. 4:**
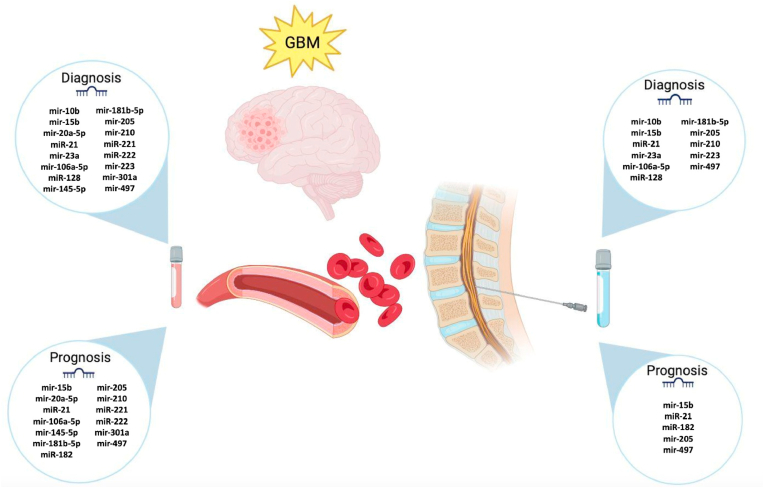
**Potential miRNAs with diagnostic and prognosis in serum and CSF liquid biopsies for GBM**. miRNAs are listed according to their potential role according to CSF or serum biopsies. Image created with www.biorender.com.

Liquid biopsies, appear as an innovative and attractive diagnostic alternative that can also serve a follow-up role to identify early recurrence. These diagnostic, and prognostic potentials in conjunction with the possibility of predicting and establishing both an adequate or inadequate therapeutic response, have been studied and associated with specific miRNAs. Some of them have a diagnostic value such as miR-21, miR-128, and miR0342–3p, (Lai et al., 2015) overlapping with prognostic ones, and drug resistance prediction abilities such as in the case of miR-21 (Huang et al., 2018; Sun et al., 2018; Kim et al., 2003). Radio resistance prediction, on the other hand, has been demonstrated to be linked to other biomarkers such as miR-128, and miR-301 (Costa-Silva et al., 2015; Liu et al., 2016). This ability to work as biomarkers was also described by André-Grégoire et al., who demonstrated higher extracellular vesicle levels in GBM patients compared to healthy controls. Aside from this, specific sets of miRNAs have proven to have a diagnostic utility such as in the case of miR-320e, miR-223, miR-23a, and miR-21, which when used as a combined ‘4-miRNA test’ has a diagnostic accuracy of 99.8%. This demonstrates that a miRNA signature may have the potential to have perfect accuracy in distinguishing glioma patients (Morokoff et al., 2020). Tumors are also able to quickly evolve and modify their molecular profiling to gain resistance to certain treatments, so having a reliable platform that allows for real-time assessment of the changes occurring in the primary tumor is highly valuable (Shankar et al., 2017). Potential miRNAs with diagnostic and prognosis in serum and CSF liquid biopsies are resumed in Fig. 4. Further work is still required to disentangle the molecular complexities of miRNAs and the functional properties of these biomarkers need further investigation to establish adequate patterns and clusters with a diagnostic potential (Ahmed et al., 2021).

All information regarding our scoping review is resumed in Table 2. Liquid biopsies in GBM hold significant potential for improving diagnosis, therapy response, tumor evolution monitoring, and prognosis. Elevated levels of microRNAs such as miR-210, miR-10 b-5p, miR-15 b-5p, and miR-182 in serum, plasma, CSF, and blood are associated with GBM and can be used to diagnose the disease and predict patient prognosis (Table 2). Conversely, decreased expression of miR-124, miR-128, miR-146 b, and miR-218, which act as tumor suppressors, correlates with poorer outcomes (Table 2). The differential expression of these microRNAs highlights their potential in personalized diagnosis and treatment, where specific microRNA profiles could guide therapeutic strategies. Advanced techniques, such as nanoparticle fluorescence quenching for detecting miR-182, enhance the accuracy and early detection capabilities, emphasizing the role of innovative diagnostic tools. The regulatory networks involving microRNAs, such as the circBFAR/miR-548 b/FoxM1 axis, further underscore the clinical relevance of these biomarkers in GBM progression and treatment response (Table 2). Overall, liquid biopsies provide a non-invasive, comprehensive approach to managing GBM, offering insights into the disease that can improve patient outcomes through tailored interventions.

**Table 2:** Characteristics of studies regarding liquid biopsies in GBM.

Authors	Year	miRNAs	Expression status	Fluid	Utility	Conclusions of study
Bustos et al. (Bustos et al., 2022)	2022	miR-21	Overexpressed	Serum/plasma, CSF	Prognosis, diagnosis, treatment response	Specific microRNAs are consistently deregulated in glioblastoma (GBM), indicating their potential as biomarkers for diagnosis, prognosis, and treatment response assessment.
		miR-10 b	Overexpressed	Serum/plasma, CSF	Prognosis, diagnosis, treatment response	
		miR-221	Overexpressed	Serum/plasma, CSF	Prognosis, diagnosis, treatment response	
		miR-10 b	Overexpressed	Serum/plasma, CSF	Prognosis, diagnosis, treatment response	
		miR-155	Overexpressed	Serum/plasma, CSF	Prognosis, diagnosis, treatment response	
		miR-182	Overexpressed	Serum/plasma, CSF	Prognosis, diagnosis, treatment response	
		miR-196 b	Overexpressed	Serum/plasma, CSF	Prognosis, diagnosis, treatment response	
		miR-7	Underexpressed	Serum/plasma, CSF	Prognosis, diagnosis, treatment response	
		miR-128	Underexpressed	Serum/plasma, CSF	Prognosis, diagnosis, treatment response	
		miR-124	Underexpressed	Serum/plasma, CSF	Prognosis, diagnosis, treatment response	
		miR-137	Underexpressed	Serum/plasma, CSF	Prognosis, diagnosis, treatment response	
		miR-218	Underexpressed	Serum/plasma, CSF	Prognosis, diagnosis, treatment response	
Ondracek et al. (Ondracek et al., 2017)	2017	miR-218	Down-regulated	Not specified	Predictive marker	The table is constructed based on data from global miRNA expression profiling of radioresistant and parental GBM cell lines, identifying miRNAs associated with radioresistance in GBM.
		miR-204	Down-regulated	Not specified	Predictive marker	
		miR-146 b-5p	Down-regulated	Not specified	Predictive marker	
		miR-31	Down-regulated	Not specified	Predictive marker	
		miR-302a	Down-regulated	Not specified	Predictive marker	
		miR-452	Not mentioned	Not specified	Predictive marker	
Bao et al. (Bao et al., 2021)	2021	miR-29b-1	Underexpressed	Not specified	Prognosis (Prolongs survival)	While miR-377's utility is mentioned in inhibiting proliferation and invasion, it's not explicitly stated whether it's specifically for GBM. Additionally, miR-670–3p is implicated in inducing ferroptosis, which could be relevant for treatment response in GBM.
		miR-129–3p	Underexpressed	Not specified	Prognosis (Prolongs survival)	
		miR-377	Not specified	Not specified	Diagnosis (Inhibits proliferation, invasion)	
		miR-670–3p	Overexpressed	Serum	Treatment response (Induces ferroptosis)	
Swellam et al. (Swellam et al., 2019)	2019	miR-221	Elevated	Blood	Diagnosis, prognosis, treatment response	Detection of circulating miR-221 and miR-222 may be used as circulating molecular marker for diagnosis and prediction of outcome for patients with GBM
		miR-222	Elevated	Blood	Diagnosis, prognosis, treatment response	
Li et al. (Li et al., 2022)	2022	miR-548 b	Underexpressed	Tissue (GBM)	Prognosis, Treatment response	miR-548 b as a Tumor Suppressor: miR-548 b acts as a tumor suppressor in GBM by inhibiting the expression of FoxM1, a gene associated with tumor progression. MiR-548 b′s underexpression in GBM tissues suggests its potential as a prognostic marker and therapeutic target.
		miR-513a-3p	Not specified	Not specified	Prognosis, Treatment response	
		miR-7	Not specified	Not specified	Prognosis, Treatment response	
		miR-17	Not specified	Not specified	Prognosis, Treatment response	
		miR-124	Not specified	Not specified	Prognosis, Treatment response	
Zhi et al. (Zhi et al., 2015)	2015	miR-20a-5p	Overexpressed	Serum	Diagnosis, Prognosis	The overexpression of miR-20a-5p, miR-106a-5p, and miR-181 b-5p was associated with advanced clinical stages of astrocytoma. Additionally, the high expression of miR-19a-3p, miR-106a-5p, and miR-181 b-5p was significantly associated with poor patient survival.
		miR-106a-5p	Overexpressed	Serum	Diagnosis, Prognosis	
		miR-181 b-5p	Overexpressed	Serum	Diagnosis, Prognosis	
		miR-19a-3p	Overexpressed	Serum	Prognosis	
Drusco et al. (Drusco et al., 2015)	2015	miR-223	Overexpressed	Cerebrospinal Fluid (CSF)	Diagnosis, Differentiating from Medulloblastoma	- The absence of miR-935 together with moderate expression of miR-451 and miR-711 could be indicative of glioblastoma or medulloblastoma. - The differential expression miR-223, miR-125 b, miR-711 could help differentiate glioblastoma from medulloblastoma.
		miR-125 b	Decreased	Cerebrospinal Fluid (CSF)	Diagnosis, Differentiating from Medulloblastoma	
		miR-711	Overexpressed	Cerebrospinal Fluid (CSF)	Diagnosis, Differentiating from Medulloblastoma	
		miR-935	Decreased	Cerebrospinal Fluid (CSF)	Diagnosis, Differentiating from Medulloblastoma	
Yang et al. (Yang et al., 2013)	2013	miR-15 b	Decreased	Serum	Diagnosis, Prognosis	- These 7 serum microRNAs were significantly decreased in patients with grade II-IV astrocytomas compared to healthy controls. - The 7-microRNA panel demonstrated high sensitivity and specificity for predicting malignant astrocytomas. - The serum levels of these microRNAs were markedly elevated after surgical resection of the tumors.
		miR-23a	Decreased	Serum	Diagnosis, Differentiating from benign astrocytoma and astrogliosis	
		miR-133a	Decreased	Serum	Diagnosis, Prognosis	
		miR-150*	Decreased	Serum	Diagnosis, Differentiating from benign astrocytoma and astrogliosis	
		miR-197	Decreased	Serum	Diagnosis, Differentiating from benign astrocytoma and astrogliosis	
		miR-497	Decreased	Serum	Diagnosis, Prognosis	
		miR-548 b-5p	Decreased	Serum	Diagnosis, Differentiating from benign astrocytoma and astrogliosis	
Zhong et al. (Zhong et al., 2019)	2019	miR-29 b	Underexpressed	Serum Exosomes	Diagnosis, Prognosis	- miR-29 b was found to be significantly underexpressed in the serum exosomes of GBM patients compared to anaplastic astrocytoma (AA) patients and healthy controls. - Serum exosomal miR-29 b levels significantly increased after surgical treatment of GBM patients. - GBM patients with low serum exosomal miR-29 b expression had significantly shorter overall survival.
Zhang et al. (Zhang et al., 2019a)	2019	miR-145–5p	Underexpressed	Serum	Diagnosis, Prognosis	- Serum levels of miR-145–5p were significantly decreased in GBM patients compared to patients with lower grade gliomas (I/II) and healthy controls. - ROC curve analysis showed that serum miR-145–5p could effectively distinguish GBM patients from the control groups. - Multivariate analysis confirmed that serum miR-145–5p expression was an independent prognostic indicator for overall survival in GBM patients
Díaz Méndez et al. (Diaz Mendez et al., 2023)	2023	miR-1-3p	Underexpressed	Serum	Diagnosis, Prognosis	- A 3-microRNA signature consisting of miR-1-3p, miR-26a-1-3p, and miR-487 b-3p was found to be differentially expressed in the serum of glioma patients compared to healthy controls. - This 3-microRNA signature was specifically downregulated in the serum of glioblastoma patients with IDH wild-type tumors compared to those with IDH-mutant tumors. - The expression and release of this 3-microRNA signature in the conditioned medium of glioma cell lines was also lower in IDH wild-type cells compared to IDH-mutant cells.
		miR-26a-1-3p	Underexpressed	Serum	Diagnosis, Prognosis	
		miR-487 b-3p	Underexpressed	Serum	Diagnosis, Prognosis	
Roth et al. (Roth et al., 2011)	2011	miR-21	Overexpressed	Blood	Diagnostic marker for GBM	Blood-derived glioblastoma-associated characteristic miRNA fingerprints may be suitable biomarkers
		miR-128	Underexpressed	Blood	Potential prognostic marker for GBM	
		miR-153	Underexpressed	Blood	Potential prognostic marker for GBM	
		miR-181 b	Underexpressed	Blood	Potential prognostic marker for GBM	
		miR-342–3p	Underexpressed	Blood	Potential prognostic marker for GBM	
Baraniskin et al. (Baraniskin et al., 2012)	2012	miR-15 b	Overexpressed/Elevated	CSF	Potential biomarker for diagnosis of glioma, including GBM	- Combining the analysis of miR-15 b and miR-21 further increased the diagnostic value for distinguishing glioma from other differential diagnoses like primary central nervous system lymphoma (PCNSL) and brain metastases - The study did not find any association between the expression levels of miR-15 b and miR-21 with glioma grading or patient survival, which the authors attributed to the small sample size of the pilot study
		miR-21	Overexpressed/Elevated	CSF	Potential biomarker for diagnosis of glioma, including GBM	
Ilhan-Mutlu et al. (Ilhan-Mutlu et al., 2012)	2012	miR-21	Overexpressed/Elevated	Plasma	Potential biomarker for prognosis and treatment response in GBM patients	
Wang et al. (Wang et al., 2012)	2012	miR-21	Overexpressed/Elevated	Plasma	Diagnostic biomarker for GBM	- miR-21, miR-128 and miR-342–3p were not significantly changed in patients with other brain tumors such as meningioma or pituitary adenoma. - The plasma levels of miR-21, miR-128 and miR-342–3p in GBM patients treated by operation and chemo-radiation almost revived to normal levels. - miR-128 and miR-342–3p were positively correlated with histopathological grades of glioma
		miR-128	Overexpressed/Elevated	Plasma	Diagnostic biomarker for GBM, Correlates with higher glioma grade	
		miR-342–3p	Overexpressed/Elevated	Plasma	Diagnostic biomarker for GBM, Correlates with higher glioma grade	
Dong et al. (Dong et al., 2014)	2014	miR-576–5p	Overexpressed	Peripheral blood	Potential biomarker for glioblastoma diagnosis	- miR-576–5p, miR-340, miR-626, miR-320, let-7g-5p, and miR-7-5p were considered as a panel or cluster of biomarkers, rather than as individual independent biomarkers.
		miR-340	Overexpressed	Peripheral blood	Potential biomarker for glioblastoma diagnosis	
		miR-626	Overexpressed	Peripheral blood	Potential biomarker for glioblastoma diagnosis	
		miR-320	Underexpressed	Peripheral blood	Potential biomarker for glioblastoma diagnosis	
		let-7g-5p	Underexpressed	Peripheral blood	Potential biomarker for glioblastoma diagnosis	
		miR-7-5p	Underexpressed	Peripheral blood	Potential biomarker for glioblastoma diagnosis	
Mohammad et al. (Mohammad et al., 2014)	2014	hcmv-miR-UL112–3p	Elevated	Plasma	Not clear	The presence of this viral microRNA in plasma of GBM patients may be a result of released exosomes or microvesicles from infected tumor cells or inflammatory cells.
Herman et al. (Herman et al., 2015)	2015	miR-21	Overexpressed	Plasma	Diagnostic, prognostic	The GBM burden is reflected in the alteration of the plasma miRNAs pattern, including viral miRNAs, representing the potential for future clinical applicatio.
		miR-124	Underexpressed	Plasma	Diagnostic, prognostic	
		miR-128	Underexpressed	Plasma	Diagnostic, prognostic	
		miR-181 b	Underexpressed	Plasma	Diagnostic, prognostic	
		miR-342–3p	Underexpressed	Plasma	Diagnostic, prognostic	
Li et al. (Li et al., 2016)	2016	miR-137	Decreased	Serum	Potential biomarker for prognosis of GBM	- Serum levels of miR-137 were significantly lower in GBM patients compared to healthy controls. - Lower serum expression levels of miR-137 were associated with poorer prognosis in GBM patients.
Akers et al. (Akers et al., 2017)	2017	miR-21	Overexpressed	CSF	Diagnostic and prognostic biomarker for GBM	Authors report a CSF miRNA signature as a “liquid biopsy" diagnostic platform for glioblastoma.
		miR-10 b	Overexpressed	CSF	Diagnostic and prognostic biomarker for GBM	
		miR-222	Overexpressed	CSF	Diagnostic and prognostic biomarker for GBM	
		miR-124	Underexpressed	CSF	Diagnostic and prognostic biomarker for GBM	
		miR-128	Underexpressed	CSF	Diagnostic and prognostic biomarker for GBM	
Yamashita et al. (Yamashita et al., 2023)	2023	miR-34 b-3p	Elevated	Plasma exosomes	Diagnostic, Potential therapeutic tool	miR-34 b-3p might have a potential as a novel diagnostic marker or a therapeutic tool for glioblastoma patients.
Barut et al. (Barut and Akdeniz, 2023)	2023	miR-22–3p	Overexpressed/Elevated	Serum	Diagnostic	Potential diagnostic biomarker for glioma with 100% specificity and 41.7% sensitivity
Park et al. (Joo Park et al., 2022)	2022	miR-21	Elevated	Blood	Diagnostic, Prognostic	miR-21 can be used as a non-invasive biomarker for diagnosing glioblastoma when tumors are inoperable or when biopsy is not feasible. MiR-21 levels before and after treatment correlate with disease progression, with increased levels indicating progressive disease and decreased levels indicating stable disease. MiR-21 levels can be monitored throughout treatment to assess response, including after surgery, chemoradiotherapy, and adjuvant temozolomide therapy.
Sippl et al. (Sippl et al., 2022)	2022	miR-181 d	Downregulated	Tumor, Plasma	Predictive biomarker for MGMT expression and treatment response to carmustine wafer implantation	miRNA-181 d seems to be a potential molecular marker that can reliably be detected in blood samples of patients with glioblastoma.
		miR-181a	Not specified	Tumor, Plasma	Prognosis/diagnosis	
Billur et al. (Billur et al., 2022)	2022	miR-582–5p	Overexpressed/Elevated	Serum	Diagnosis	- Serum levels of miRNA-582–5p and miRNA-363 were significantly upregulated in glioblastoma (GBM) patients compared to healthy controls. - High levels of miRNA-582–5p (fold change 2.86, p < 0.0001) and miRNA-363 (fold change 3.51, p < 0.0001) were significantly associated with glioblastoma (GBM)
		miR-363	Overexpressed/Elevated	Serum	Diagnosis	
Vojdani et al. (Vojdani et al., 2021)	2021	miR-34a	Underexpressed/Decreased	Serum, Tissue	Diagnosis	Dysregulation of the EGFR gene and miR-34a in serum samples of GBM patients promises the emergence of non-invasive biomarkers for early detection of GBM.
Rahmati et al. (Rahmati et al., 2021)	2021	miR-330–3p	Decreased	Tissue, Serum	Prognostic biomarker	mir-330–3p and mir-485–5p could be potential biomarkers in GBM.
		miR-485–5p	Decreased	Tissue, Serum	Prognostic biomarker	
Swellam et al. (Swellam et al., 2021)	2021	miR-17–5p	Decreased	Serum	Prognostic and treatment response marker	Detection of serum miR-17–5p, miR-125 b, and miR-221 aids in the prediction of prognosis and response to treatment strategy for GBM patients.
		miR-125 b	Decreased	Serum	Prognostic and treatment response marker	
		miR-221	Decreased	Serum	Prognostic and treatment response marker	
Fleischmann et al. (Fleischmann et al., 2020)	2020	hsa-let-7a-5p	Not specified	Blood plasma	Prognosis	Blood plasma based risk stratification through the 4-miRNA risk score shows strong differences in progression-free survival of glioblastoma patients.
		hsa-let-7a-5p	Not specified	Blood plasma	Prognosis	
		hsa-miR-125a-5p	Not specified	Blood plasma	Prognosis	
		hsa-miR-615–5p	Not specified	Blood plasma	Prognosis	
Gareev et al. (Gareev et al., 2020)	2020	miR-21	Overexpressed/Elevated	CSF	Diagnosis	- The level of expression of miR-128 was seen to increase in the blood of patients with GBM, while it was significantly decreased in GBM tissues. - miR-128 levels correlated positively with the histopathological classes of gliomas, including GBM. - The activity of miR-342–3p was shown to decrease in the blood of patients with GBM. - Similar to miR-128, miR-342–3p levels correlated positively with the histopathological classes of gliomas.
		miR-128	Increased	Blood	Diagnosis	
		miR-342–3p	Decreased	Blood	Diagnosis	
		miR-221/222	Increased	Plasma	Prognosis	
Kopkova et al. (Kopkova et al., 2019a)	2019	miR-10 b	Overexpressed	CSF	Prognosis	- Patients with high miR-10 b/miR-196 b levels had median overall survival of 9 months, compared to 16.5 months in patients with low levels - miR-196a is associated with glioma progression
		miR-196 b	Overexpressed	CSF	Prognosis	
		miR-196a	Overexpressed	Tissue	Monitoring	
Kopkova et al. (Kopkova et al., 2019b)	2019	miR-21	Overexpressed	Serum, CSF, Tissue	Not mentioned	There is potential of CSF miRNAs to be useful biomarkers in brain tumors, including GBM.
		miR-10 b	Overexpressed	Serum, Tissue	Not mentioned	
		miR-221	Overexpressed	Serum, Tissue	Not mentioned	
		miR-128	Underexpressed	Serum, Tissue	Not mentioned	
		miR-155	Overexpressed	Serum, Tissue	Not mentioned	
ParvizHamidi et al. (ParvizHamidi et al., 2019)	2019	miR-21	Overexpressed/Elevated	Serum	Potential diagnostic biomarker	- miR-21 and miR-26a were significantly upregulated in the serum of GBM patients compared to non-cancerous controls - miR-128 was found to be significantly downregulated in the plasma of GBM patients compared to controls. - The serum levels of miR-21, miR-26a, and miR-128 were reduced in post-operative samples compared to pre-operative samples, with the decrease being significant for miR-26a.
		miR-26a	Overexpressed/Elevated	Serum	Potential diagnostic biomarker	
		miR-128	Underexpressed/Decreased	Plasma	Potential diagnostic biomarker	
Yuan et al. (Yuan et al., 2019)	2019	miR-365	Decreased	Serum, Tissue	Diagnosis	- The serum expression of miR-365 was downregulated in glioblastoma patients compared to healthy controls. - The expression of miR-365 was also decreased in glioblastoma tissue samples compared to adjacent normal tissues. - Overexpression of miR-365 suppressed glioblastoma cell proliferation, migration, and epithelial-to-mesenchymal transition. - PAX6 was identified as a direct target gene of miR-365 in glioblastoma cells
Manterola et al. (LAB–OMICS AND PROGNOSTIC MARKERS, 2012)	2012	miR-320	Elevated	Serum	Diagnosis, Prognosis	Small non-coding RNAs isolated from the microvesicles of the serum of GBM patients could serve as a noninvasive predictor for diagnosis of GBM patients
		miR 574–3p	Elevated	Serum	Diagnosis, Prognosis	
Ray et al. (Ray and Akolkar, 2016)	2016	miR-27a	Elevated	Blood	Diagnosis, Monitoring	Liquid biopsy can play an important role in the diagnosis of patients with gliomas and reduce the under reporting of high grade gliomas caused by tumor heterogeneity.
		miR-210	Elevated	Serum, CSF	Diagnosis, Monitoring	
		miR-124	Elevated	Blood	Diagnosis, Monitoring	
Zhang et al. (Zhang et al., 2019b)	2019	miR-100	Decreased	Serum	Diagnosis, Prognosis	Serum miR-100 might serve as promising biomarker for GBM diagnosis and prognosis.
		miR-106a	Decreased	Not specified	Prognosis (poor)	
		miR-485–3p	Decreased	Not specified	Prognosis (poor)	
		miR-328	Decreased	Not specified	Prognosis (poor)	
		miR-137	Decreased	Not specified	Prognosis (poor)	
		miR-196 b	Increased	Not specified	Diagnosis	
		miR-15 b	Increased	Not specified	Diagnosis	
Zeng et al. (Zeng et al., 2018)	2018	miR-151a	Decreased	CSF, Serum	Prognosis, Diagnosis, Treatment response	- Lower expression of miR-151a was associated with worse overall survival and progression-free survival in GBM patients treated with TMZ. - Restoring miR-151a expression in TMZ-resistant cells sensitized them to TMZ treatment. - Levels of miR-151a in CSF-derived exosomes could serve as a “liquid biopsy" biomarker to predict chemotherapy response in GBM patients
Gao et al. (Gao et al., 2016)	2016	miR-30e	Upregulated	Serum	Postoperative monitoring	Four miRNAs, namely, miR-26 b, miR-30e, miR-129–3p, and miR-206, were selected on the basis of previous and present findings. A low miR-30e expression level corresponded to prolonged survival.
Charbit et al. (Charbit et al)	2018	miR-10 b	Upregulated	Serum	Treatment response monitoring	Treatment response here was to bevacizumab specifically
		miR-21	Upregulated	Serum	Treatment response monitoring	
Cai et al. (Cai et al., 2018)	2018	miR-148a	Overexpressed/Elevated	Serum exosomes	Diagnosis	- Circulating exosomal miR-148a levels were significantly higher in serum from GBM patients compared to healthy volunteers - Inhibition of miR-148a suppressed cell proliferation and metastasis in T98G GBM cells
Wang et al. (Wang et al., 2017)	2017	miR-485–3p	Decreased	Serum	Prognosis	No significant correlation was found between survival rates (PFS and OS) and the expression levels of miR-451a and miR-4298 in GBM patients (both P > 0.05, Fig. 2A and C)
		miR-451a	Not mentioned	Serum	–	
		miR-4298	Not mentioned	Serum	–	
Zhao et al. (Zhao et al., 2017b)	2017	miR-106a-5p	Overexpressed	Serum	Prognostic biomarker for overall survival and disease-free survival	- High levels of miR-106a-5p, miR-182, miR-222–3p, and miR-20a-5p were associated with poor prognosis (decreased survival). - Low levels of miR-145–5p were associated with improved prognosis (increased survival).
		miR-182	Overexpressed	Serum	Prognostic biomarker for overall survival and disease-free survival	
		miR-145–5p	Underexpressed	Serum	Prognostic biomarker for overall survival and disease-free survival	
		miR-222–3p	Overexpressed	Serum	Prognostic biomarker for disease-free survival	
		miR-20a-5p	Overexpressed	Serum	Prognostic biomarker for disease-free survival	
Chen et al. (Chen et al., 2017b)	2017	miR-203	Decreased	Serum	Prognostic indicator of poor overall survival and progression-free survival	- miR-203: Patients with lower serum miR-203 expression had poorer overall survival (OS) and progression-free survival (PFS) - miR-663: GBM patients with lower miR-663 levels suffered an unfavorable clinical outcome compared to those with higher miR-663 expression - For the 5-microRNA signature including miR-222, miR-132, miR-129, miR-145 and miR-20a, the file states it was associated with clinical outcome and chemoresistance in GBM patients with MGMT promoter methylation, but does not specify whether the outcome was better or worse.
		miR-222	Increased	Serum	Associated with clinical outcome and chemoresistance in GBM patients with MGMT promoter methylation	
		miR-132	Increased	Serum	Associated with clinical outcome and chemoresistance in GBM patients with MGMT promoter methylation	
		miR-129	Increased	Serum	Associated with clinical outcome and chemoresistance in GBM patients with MGMT promoter methylation	
		miR-145	Decreased	Serum	Associated with clinical outcome and chemoresistance in GBM patients with MGMT promoter methylation	
		miR-20a	Increased	Serum	Associated with clinical outcome and chemoresistance in GBM patients with MGMT promoter methylation	
		miR-663	Decreased	Not specified	Prognosis (poor)	

### 3.8 The miRNA genome is a treasure for GBM treatment

A profound understanding of diverse genetic mechanisms and their interactions is the future of diagnosing and treating GBM. This may involve utilizing diagnostic biomarkers present within the body and delivering personalized delivery of drugs via nanoparticles. Such an approach can offer a less invasive and precise alternative to surgery in some specific scenarios in the future. Once integrated with neuronal differentiation modeling and the intricate networks of miRNAs, the subsequent challenge is to identify specific epigenetic targets for GBM therapy and advance strategies for novel drug discovery. The objective of miRNA-based glioma therapy is to halt tumor progression and trigger apoptosis in malignant cells, restoring normal cellular pathway functions. The efficacy of miRNA-based therapy is evaluated by assessing the glioma cell population or metabolism post-treatment using various assays. (Jimenez-Morales et al., 2022) miRNAs present a promising and innovative treatment avenue for GBM. However, their clinical implementation faces significant challenges, particularly related to the blood-brain barrier and miRNA stability in body fluids (Jimenez-Morales et al., 2022).

Combining a miRNA-21 inhibitor or miRNA-7 mimic with TMZ shows great promise as a strategy to potentially overcome TMZ resistance mechanisms. Both the miRNA-21 inhibitor and miRNA-7 mimic have been recognized as crucial regulatory elements associated with the four most significant cancer hallmarks related to therapy (Jimenez-Morales et al., 2022): 1) replicative immortality, 2) invasion and migration, 3) resistance to cell death, and angiogenesis induction (Rupaimoole and Slack, 2017). For example, the following microRNAs have been found to intervene in cancer hallmarks inhibiting the following processes: 1) cell cycle arrest (miRNA-10 b and miRNA-21), 2) metastasis inhibition (miRNA-10 b and miRNA-21), 3) apoptosis recovery (miRNA-9, miRNA-10 b, miRNA-21, miRNA-221, miRNA-222), and 4) angiogenesis inhibition (miRNA-21) (Jimenez-Morales et al., 2022). By using these miRNA-based approaches in conjunction with TMZ, there is a possibility of enhancing the effectiveness of GBM treatment and addressing the challenges posed by TMZ resistance mechanisms. The targeted regulation of these miRNAs holds the potential to improve outcomes and provide a novel approach to tackling glioma therapy.

### 3.9 Future directions

The constantly evolving field of neuro-oncology has been integrating the molecular profiling of CNS tumors into clinical practice. The importance of approaching these tumors from different molecular perspectives, especially in highly morbid tumors such as GBM, is crucial for achieving better outcomes. The inclusion of miRNAs into the neuro-oncological management of CNS tumors shows great promise as their role has been elucidated in recent studies (Anthiya et al., 2018; Beylerli et al., 2023). These molecules open new ways for developing molecular biomarkers and novel treatments that could be integrated into clinical practice. Furthermore, combining the histologic, imaging, and molecular methods of this disease asserts a more complete and comprehensive way of approaching CNS tumors. Expanding the potential applications of molecular tools such as miRNAs with the use of less-invasive diagnostic techniques such as liquid biopsies, could improve the individualization of patients regarding the diagnosis, management, and prognosis of aggressive tumors. The need for continuous research into this highly morbid disease makes necessary continuous efforts for new and novel treatments.

Finally, there is a big need to provide physicians with accurate tools. As mentioned before, many miRNAs work together and overlap in different mechanisms of action. Consequently, the development of signatures or clusters may help to establish new rapid and accurate diagnostic and prognostic tools for GBM. Also, the detection and correlation between tumoral and serum or CSF miRNAs is still debatable and needs further investigation.

## 4 Critical view

The manuscript presents a comprehensive review focusing on the current state of the art regarding microRNA (miRNA) and non-invasive techniques for miRNA detection in glioblastomas (GBMs), with a specific emphasis on liquid biopsies in cerebrospinal fluid and serum. This topic holds significant relevance in the context of current advancements in molecular diagnostics and treatment strategies aimed at enhancing targeted therapies for GBM in clinical practice.

What distinguishes our review from the existing literature is its concentrated focus on the utilization of miRNAs as biomarkers in liquid biopsies for GBM detection. While previous studies have explored various molecular diagnostic approaches for GBM, our manuscript places particular emphasis on understanding the molecular aspects of miRNAs and the potential of miRNAs in liquid biopsies as a less invasive means of diagnosis, management, and prognosis for GBMs. The significance of our work lies in its contribution to the evolving field of neuro-oncology, where molecular profiling of central nervous system (CNS) tumors is becoming increasingly integrated into clinical practice. Furthermore, the inclusion of miRNAs in the management of CNS tumors shows great promise, as their roles have been elucidated in recent studies. Expanding the applications of molecular tools such as miRNAs, particularly through less invasive techniques like liquid biopsies, has the potential to enhance the individualization of patient care, ranging from diagnosis to prognosis and treatment selection for aggressive tumors like GBM.

In summary, our manuscript offers a unique perspective on the current state of the art regarding miRNAs and GBM and the role of liquid biopsies in GBM, contributing to the advancement of molecular diagnostics and personalized medicine in neuro-oncology. We believe that this review fills a critical gap in the literature and has the potential to significantly benefit the current knowledge and future clinical management of GBM patients.

## 5 Conclusions

MiRNAs have been demonstrated to play a potential tool in the diagnosis, treatment, and prognosis of GBM. New strategies for rapid and accurate detection like liquid biopsies may be a minimally invasive solution to provide sequential information before and after treatments, improving the diagnostic and prognostic information of these tumors. MiRNAs may work as signatures or clusters and further investigation to develop new diagnostic markers are needed. GBM remains a fatal and heterogeneous tumor that requires intense research to improve survival, miRNAs seem to be promissory and remain a remarkable research topic.

## Credit author statement

Conceptualization: Ordonez-Rubiano EG, Parra-Medina R. Data curation: Ordonez-Rubiano EG, Parra-Medina R, Payán-Gómez C. Formal analysis: Cómbita A, Hakim F, Gómez-Amarillo DF, Ordonez-Rubiano EG, Parra-Medina R, Payán-Gómez C, Salazar AF, Shelton WJ, Rincón-Arias N, Espinosa S. Funding acquisition: N/A. Investigation: Cómbita A, Hakim F, Gómez-Amarillo DF, Ordonez-Rubiano EG, Parra-Medina R, Payán-Gómez C, Salazar AF, Shelton WJ, Rincón-Arias N, Espinosa S. Methodology: Ordonez-Rubiano EG, Parra-Medina R, Project administration: Ordonez-Rubiano EG, Parra-Medina R, Cómbita A. Resources: N/A. Software: N/A. Supervision: Ordonez-Rubiano EG, Parra-Medina R, Cómbita A, Hakim F. Validation: Cómbita A, Hakim F, Gómez-Amarillo DF, Ordonez-Rubiano EG, Parra-Medina R, Payán-Gómez C, Baldoncini M, Luzzi S, Salazar AF, Shelton WJ, Rincón-Arias N, Espinosa S. Visualization: N/A. Roles/Writing - original draft: Cómbita A, Hakim F, Gómez-Amarillo DF, Ordonez-Rubiano EG, Parra-Medina R, Payán-Gómez, Salazar AF, Shelton WJ, Rincón-Arias N, Espinosa S. Writing - review & editing: Cómbita A, Hakim F, Gómez-Amarillo DF, Ordonez-Rubiano EG, Parra-Medina R, Payán-Gómez C Baldoncini M, Luzzi S, Salazar AF, Shelton WJ, Rincón-Arias N, Espinosa S.

## 6 AI disclosure

During the preparation of this work, the author(s) used TextCortex in order to improve Table 2 of the scoping review and provide useful information regarding liquid biopsies in GBM given the large amount of varied information in the literature. After using this tool/service, the authors reviewed and edited the content as needed and take full responsibility for the content of the publication.

## Declaration of competing interest

The authors declare that they have no known competing financial interests or personal relationships that could have appeared to influence the work reported in this paper.

## Data Availability

No data was used for the research described in the article.
